# Unemployment and Depression Among Emerging Adults in 12 States, Behavioral Risk Factor Surveillance System, 2010

**DOI:** 10.5888/pcd12.140451

**Published:** 2015-03-19

**Authors:** Robin E. McGee, Nancy J. Thompson

**Affiliations:** Author Affiliation: Nancy J. Thompson, Emory University, Atlanta, Georgia.

## Abstract

**Introduction:**

The high rate of unemployment among emerging adults (aged 18 to 25 years) is a public health concern. The risk of depression is higher among the unemployed than among the employed, but little is known about the relationship between unemployment and mental health among emerging adults. This secondary data analysis assessed the relationship between unemployment and depression among emerging adults.

**Methods:**

Data from the 2010 Behavioral Risk Factor Surveillance System (BRFSS) were analyzed. Responses to the Patient Health Questionnaire-8 provided data about the prevalence of depression. Bivariate relationships were assessed using χ^2^ tests, and multivariable adjusted odds ratios were calculated with logistic regressions. Sociodemographic variables were sex, race/ethnicity, marital status, and education. In addition, logistic regression models adjusted for health insurance status, disability, smoking, and body mass index. The analyses were completed using SAS 9.3 survey procedures to account for the complex sampling design.

**Results:**

Almost 12% of emerging adults were depressed (PHQ-8 ≥10) and about 23% were unemployed. Significantly more unemployed than employed emerging adults were classified with depression. In the final model, the odds of depression were about 3 times higher for unemployed than employed emerging adults.

**Conclusion:**

The relationship between unemployment and depression is significant among emerging adults. With high rates of unemployment for this age group, this population may benefit from employment- and mental-health–focused interventions.

## Introduction

Depressive disorders are among the most common mental health problems (1). As a leading cause of disability (2), depression is related to reduced quality of life and increased risk for physical health problems (3). Although depression has substantial consequences throughout the lifespan, depression during emerging adulthood, the period of transition from adolescence to adulthood (4), influences long-term consequences through recurrent depressive episodes (5) and worse socioeconomic outcomes (6). Annually, 8.3% of adults aged 18 to 25 report having had at least 1 major depressive episode (7).

Although many factors contribute to depression, unemployment is consistently associated with high rates of depression among adults (8,9). Unemployment may contribute to depression because of losses in social contact and status or stress related to income loss (10). For emerging adults, long experiences of unemployment increase the likelihood of experiencing depression throughout the transition (11).

The high unemployment rate among emerging adults, around 20% in 2010 (12), is a substantial public health problem (13). The potential situational stressor of being unemployed and the developmental stressor of transitioning to young adulthood (14) may combine to increase experiences of depression. However, few studies relating unemployment and depression focus on emerging adults. For example, Brown et al (15) excluded those aged 18 to 25 in their study examining frequent mental distress and unemployment, and Galambos et al (11) did not measure clinically significant depression in their examination of depressive symptoms among recent graduates.

Mental health among emerging adults who are not students is an underresearched topic (16). This study sought to assess the relationship between unemployment and depression in a sample of emerging adults who did not identify themselves as students. We hypothesized that the prevalence of depression would be higher among unemployed compared with employed emerging adults while controlling for potentially confounding covariates.

## Methods

We analyzed data from the 2010 Behavioral Risk Factor Surveillance System (BRFSS) (17). The BRFSS is a national survey that assesses health risk behaviors among the noninstitutionalized US adult population (aged ≥18 years). State health departments work with the Centers for Disease Control and Prevention to collect BRFSS data. A core set of questions is asked in all 50 states as well as the District of Columbia, Puerto Rico, and the US Virgin Islands. In addition, state health departments may choose to include supplemental modules that ask questions on specific topics, such as depression. In 2010, 12 states (Arizona, Georgia, Hawaii, Indiana, Louisiana, Mississippi, Missouri, Nevada, South Carolina, Vermont, Wisconsin, and Wyoming) included an optional module assessing the prevalence of anxiety and depression. The overall response rates for these states ranged from 25.6% to 49.2% (18).

### Population

The sample for this study was restricted to emerging adults aged 18 to 25, inclusive. Only individuals reporting that they were employed, self-employed, unemployed more than 1 year, or unemployed less than 1 year were included in the sample. Students, homemakers, retirees, and those unable to work were excluded from the sample. Of the selected sample (n = 1,703), 89.5% provided responses to each question included in the analyses, resulting in a final sample of 1,525 (Figure).

**Figure:**
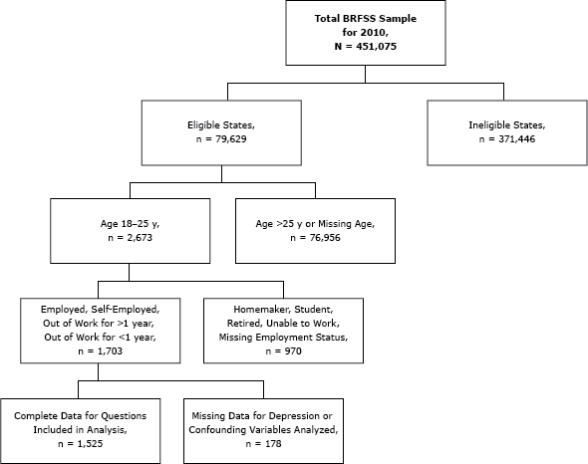
Selection of sample in analyses of unemployment and depression among emerging adults aged 18 to 25 years in 12 states, Behavioral Risk Factor Surveillance System, 2010.

### Measures

The dependent variable was depression. A score for depression was calculated based on responses to 8 questions from the Patient Health Questionnaire (PHQ-8) (19,20). The questions ask about depressive symptoms over the previous 2 weeks. For example, “Over the last 2 weeks, how many days have you had little interest or pleasure in doing things?” For each question, individuals received a score between 0 and 3, depending on the number of days they reported having the specific depressive symptom. Those who responded that they had had a depressive symptom for 0 days or 1 day received a score of 0. Those who responded that they had had a depressive symptom for 2 to 6 , 7 to 11, or 12 to 14 days, inclusive, received scores of 1, 2, or 3, respectively. The scores for each question were summed. By using criteria from other BRFSS studies of the PHQ-8, we classified those who had a total score greater than or equal to 10 as depressed (19).

The main independent variable of interest was unemployment status. Those who responded that they had been out of work for less than 1 year or more than 1 year were classified as unemployed. Respondents who indicated they were “employed for wages” or “self-employed” were classified as employed.

In addition, potentially confounding variables were included in the analyses because of their association with both depression and employment status. These variables were disability status, smoking status, and body mass index (BMI), and health insurance status as well as sociodemographic variables. These variables were selected based on previous work by Brown et al (15) examining the relationship between unemployment and mental distress among adults.

Disability status was measured by creating 3 categories of respondents using 2 questions in the 2010 BRFSS. The questions ask “Are you limited in any way in any activities because of physical, mental, or emotional problems?” and “Do you now have any health problems that require you to use special equipment, such as a cane, a wheelchair, a special bed, or a special telephone? (Include occasional use or use in certain circumstances).” Respondents who indicated that yes, they were limited in activities but that they did not require any special equipment were 1 group (no equipment needed). Respondents who indicated that yes, they were limited in activities and they needed special equipment were another group (equipment needed). The reference group responded no to both questions about disability.

Smoking status was assessed by categorizing responses to the question “Do you now smoke cigarettes every day, some days, or not at all?” Individuals who responded every day or some days were classified as smokers. Individuals who responded not at all were classified as nonsmokers.

Respondents were classified into 3 BMI categories based on a calculated variable that divided respondents’ weight in kilograms (kg) by their height in meters squared (m^2^). Those with BMIs less than 25.0 were categorized as normal weight or underweight, those with BMIs between 25.0 and 29.9 were categorized as overweight, and those with BMIs greater than or equal to 30.0 were categorized as obese.

Health insurance status was assessed by categorizing responses to the question “Do you have any kind of health care coverage, including health insurance, prepaid plans such as HMOs, or government plans such as Medicare?” Response options were yes, no, or don’t know/not sure. Those who responded yes were classified as individuals with health insurance and those who responded no were classified as individuals without health insurance. A don’t know/not sure response was considered a nonresponse.

All of the multivariable analyses adjusted for sociodemographic variables. For sex, female was the reference category. Race/ethnicity options were white, black or African American, other, and Hispanic or Latino, with white as the reference category. Marital status options were married or not married, with not married as the reference category. Education options were less than a high school degree or high school degree or higher, with high school degree or higher as the reference category.

### Analyses

First, the frequency of each variable was assessed. Then, the bivariate relationships were calculated with χ^2^ analyses. Multivariable odds ratios were calculated with logistic regressions. Three logistic regression models were fit to the data to examine how the relationship between depression and being unemployed changed with the addition of potentially confounding variables. The first model examined the relationship between unemployment and depression while controlling for health insurance status and sociodemographic variables. The sociodemographic variables were sex, race/ethnicity, marital status, and education level. The second model added disability status to the analysis. The last model adjusted for smoking and BMI in addition to the sociodemographic and disability variables. The analyses were completed using weighted data to account for the complex sample with SAS version 9.3 (SAS Institute, Inc).

## Results

Almost 12% of emerging adults were depressed (PHQ-8 ≥10), and about 23% were unemployed. Most of the weighted population was male (58.6%) and white (64.5%). Most had received a high school degree or higher (87.3%). Table 1 provides the descriptive statistics for each of the variables in the analyses.

**Table 1:** Description and Frequency of Variables Studied Among Emerging Adults Aged 18 to 25 Years (n = 1,525), Behavioral Risk Factor Surveillance System, 2010

Variable	Measures	Sample Size	Weighted %
**Depression (DV)**	PHQ-8 scores ≥10	196	11.9
	PHQ-8 scores <10	1,329	88.1
**Employment status**	**Unemployed**	344	23.0
	<1 year	207	14.5
	>1 year	137	8.5
	**Employed**	1,181	77.0
	Employed for wages	1,086	71.0
	Self-employed	95	6.0
**Sex**	Female	827	41.4
	Male	698	58.6
**Race/ethnicity**	Black or African American	326	17.7
	Hispanic or Latino	129	9.8
	Other (American Indian/Alaskan Native, Asian, Native Hawaiian or other Pacific Islander, multiple non-Hispanic/Latino races)	197	8.0
	White	873	64.5
**Marital status**	Married	251	13.3
	Not married	1,274	86.7
**Education**	Less than high school degree	176	12.7
	High school degree or higher	1,349	87.3
**Disability**	Yes, no equipment needed	153	10.6
	Yes, equipment needed	11	1.0
	No	1,361	88.4
**Smoking status**	Smoker	421	27.7
	Nonsmoker	1,104	72.3
**Health insurance status**	No	521	36.9
	Yes	1,004	63.1
**BMI, kg/m^2^**	≥30.0	337	22.4
	25.0–29.9	405	25.0
	<25.0	783	53.6

Abbreviations: BMI, body mass index; DV, dependent variable; PHQ-8, Patient Health Questionnaire-8.

In bivariate analyses, depression was more likely among unemployed emerging adults (23.4%) compared with employed emerging adults (8.4%) (χ^2^ = 27.8, *P* < .001) (Table 2). Additionally, depression was significantly higher for the unemployed for numerous potentially confounding variables. Females were more likely to report depression (15.0%) compared with males (9.6%) (χ^2^ = 4.8, *P* = .03). About 22.2% of emerging adults without a high school degree reported depression compared with 10.4% of those with at least a high school degree (χ^2^ = 11.2, *P* < .001). Those without health insurance (17.7%) reported depression more frequently compared with those with health insurance (8.5%) (χ^2^ = 13.7, *P* < .001). Those reporting a disability but not needing equipment (36.6%) were more likely to report depression compared with those with a disability and needing equipment (14.2%) and those without a disability (8.9%) (χ^2^ = 54.2, *P* < .001). Smokers (20.8%) were more likely to report depression compared with nonsmokers (8.4%) (χ^2^ = 20.4, *P* < .001). Finally, 16.8% of respondents classified as obese reported depression, which was significantly more than those who were categorized as overweight (7.9%) and normal weight or underweight (11.7%) (χ^2^ = 7.3, *P* = .03).

**Table 2:** Characteristics of Unemployed Emerging Adults Aged 18 to 25 Years by Depression and Employment Status, Behavioral Risk Factor Surveillance System, 2010

Variable	n (Weighted %)^a^	
	PHQ-8 Scores ≥10	Unemployed
**Unemployed**	77 (23.4)^b^	NA
<1 year	47 (23.0)	
>1 year	30 (24.3)	
**Employed**	119 (8.4)	
Employed for wages	103 (8.1)	
Self-employed	16 (11.9)	
**Sex**		
Female	120 (15.0)^c^	176 (20.9)
Male	76 (9.6)	168 (24.5)
**Race/ethnicity**		
Black or African American	45 (13.0)	108 (37.0)^b^
Hispanic or Latino	12 (6.4)	33 (22.5)
Other	23 (12.9)	39 (19.5)
White	116 (12.3)	164 (19.7)
**Marital status**		
Married	35 (16.7)	31 (12.8)^d^
Not married	161 (11.1)	313 (24.6)
**Education level**		
Less than high school degree	39 (22.2)^b^	77 (38.7)^b^
High school degree or higher	157 (10.4)	267 (20.7)
**Insurance status**		
No health insurance	93 (17.7)^b^	167 (32.9)^b^
Health insurance	103 (8.5)	177 (17.2)
**Disability**		
Yes, no equipment needed	56 (36.6)^b^	45 (28.0)
Yes, equipment needed	3 (14.2)	2 (4.8)
No	137 (8.9)	297 (22.6)
**Smoking status**		
Smoker	98 (20.8)^b^	136 (31.9)^b^
Nonsmoker	98 (8.4)	208 (19.6)
**BMI, kg/m^2^**		
≥30.00	57 (16.8)^c^	85 (24.6)
25.0–29.9	45 (7.9)	81 (23.1)
<25.0	94 (11.7)	178 (22.3)

Abbreviations: BMI, body mass index; NA, not applicable; PHQ-8, Patient Health Questionnaire-8.

a χ^2^ tests were used to assess significant differences.

b *P* < .001.

c *P* < .05.

d *P* < .01.

Being unemployed was associated with other independent variables. Blacks or African Americans (37.0%) were significantly more likely to report being unemployed compared with Hispanics or Latinos (22.5%), members of other races/ethnicities (19.5%), and whites (19.7%) (χ^2^ = 18.6, *P* < .001). Those without a high school degree (38.7%) were significantly more likely to report being unemployed compared with those with a high school degree or higher (20.7%) (χ^2^ = 15.1, *P* < .001). Emerging adults who were not married (24.6%) were more likely to report being unemployed compared with those who were married (12.8%) (χ^2^ = 7.18, *P* = .007). Emerging adults without health insurance (32.9%) were more likely to report being unemployed compared with those with health insurance (17.2%) (χ^2^ = 21.0, *P* < .001). Additionally, 31.9% of smokers reported being unemployed compared with 19.6% of nonsmokers (χ^2^ = 11.0, *P* < .001).

Three multivariable logistic regression models were fit to the data (Table 3). The first model examined the relationship between unemployment and depression while controlling for sociodemographic variables as potentially confounding variables. This model was significant (χ^2^[8], 69.96, *P* < .001). In this model, being unemployed increased the odds of depression (OR, 3.25; 95% confidence interval [CI], 1.93–5.47). Among the sociodemographic variables, females (OR, 2.04), those who were married (OR, 2.28), those with less than a high school degree (OR, 1.97), and those without health insurance (OR, 2.35; 95% CI, 1.48–3.73) were at increased odds for depression. Respondents who were of Hispanic or Latino race/ethnicity were at decreased odds of depression (OR, 0.34; 95% CI, 0.13–0.87).

**Table 3:** Odds of Depression Among Emerging Adults Aged 18 to 25 Years, Behavioral Risk Factor Surveillance System, 2010

Variable	Odds Ratio (95% Confidence Interval)		
	Model 1	Model 2	Model 3
**Unemployed**	3.25 (1.93–5.47)^a^	3.34 (1.92–5.80)^a^	3.17 (1.79–5.59)^a^
**Female**	2.04 (1.29–3.23^b^	1.99 (1.21–3.28)^b^	1.99 (1.18–3.38)^c^
**Race/ethnicity**			
Black or African American	0.76 (0.42–1.36)	0.91 (0.50–1.65)	0.97 (0.54–1.75)
Hispanic or Latino	0.34 (0.13–0.87)^c^	0.36 (0.14–0.93)^c^	0.36 (0.13–1.00)^c^
Other	1.37 (0.49–3.78)	1.59 (0.55–4.55)	1.66 (0.56–4.90)
White	1 [Reference]	1 [Reference]	1 [Reference]
**Married**	2.28 (1.27–4.10)^b^	2.25 (1.21–4.17)^c^	2.31 (1.24–4.28)^b^
**Less than high school degree**	1.97 (1.10–3.51)^c^	1.84 (1.02–3.29)^c^	1.50 (0.81–2.78)
**No health insurance**	2.35 (1.48–3.73)^a^	2.29 (1.40–3.75)^b^	1.92 (1.16–3.17)^c^
**Disability**			
Yes, no equipment needed	NA	5.60 (3.13–10.02)^a^	5.28 (2.80–9.95)^a^
Yes, equipment needed		4.20 (0.78–22.77)	4.87 (0.92–25.85)
No		1 [Reference]	1 [Reference]
**Smoker**	NA	NA	2.38 (1.43–3.95)^b^
**BMI kg/m^2^**			
≥30.00	NA	NA	1.37 (0.77–2.41)
25.0–29.9			0.65 (0.36–1.16)
<25.0			1 [Reference]
**Model significance**	*df*(8), 69.96, *P* < .001	*df*(10) 116.59, *P* < .001	*df*(13) 121.42, *P* < .001

Abbreviation: BMI, body mass index; NA, not applicable because variable was not used in the model.

a *P* < .001.

b *P* < .01.

c *P* < .05.

The second model added disability status as a potentially confounding variable. The model was significant (χ^2^[10], 116.59, *P* < .001). Being unemployed remained significantly associated with depression (OR, 3.34; 95% CI, 1.92–5.80). The same sociodemographic variables, females (OR, 1.99; 95% CI, 1.21–3.28), being married (OR, 2.25; 95% CI, 1.21–4.17), having less than a high school degree (OR, 1.84), and not having health insurance (OR, 2.29) remained significantly associated with the odds of depression. Disability without needing equipment also significantly increased the odds of depression (OR, 5.60; 95% CI, 3.13–10.02), but disability needing equipment did not (OR, 4.20; 95% CI, 0.78–22.77). Hispanic or Latino race/ethnicity remained protective against depression (OR, 0.36; 95% CI, 0.14–0.93).

The final model added smoking status and BMI. The model was significant (χ^2^[13], 121.42, *P* < .001). With all the potentially confounding variables included in the final model, unemployed status was associated with more than 3 times the odds of depression compared with those who were employed (OR, 3.17; 95% CI, 1.79–5.59). Women (OR, 1.99), those without health insurance (OR, 1.92), and smokers (OR, 2.38) had about 2 times the odds of depression. Those who were married were also at increased odds of depression (OR, 2.31). Disability status for those not needing equipment (OR, 5.28) also significantly increased the odds of depression, but disability status for those needing equipment (OR, 4.87; 95% CI, 0.92–25.85) did not significantly increase the odds of depression.

## Discussion

The high rate of unemployment among emerging adults is a public health problem (13). In light of this elevated prevalence, the association between poor health outcomes, including mental health, and unemployment warrants attention from individuals, families, and policy makers. The findings from this study suggest that unemployed emerging adults have 3 times greater odds of reporting depression compared with employed emerging adults, even when controlling for potentially confounding variables such as disability status.

These results suggest that unemployment among emerging adults may contribute to depression, which is consistent with research focusing on unemployed adults (8). Developmental factors, such as uncertainty related to the transition to adulthood and changes in social relationships and support structures (4,16), may contribute to different experiences of unemployment among emerging adults compared with older adults. Depression among unemployed emerging adults may be associated with stress because of delays in achieving development goals related to the transition to adulthood, including identity formation through exploring work opportunities (21). Alternatively, some may experience stressors similar to those of older adults, including stigma related to unemployment or material deprivation (22). Examining mediators between unemployment and depression could inform whether the factors that contribute to depression are similar for emerging adults and older adults. Furthermore, other social factors that contribute to mental health outcomes, such as socioeconomic status (15), could moderate this relationship (8). Additional research examining the factors that contribute to depression among unemployed emerging adults is warranted.

Reducing the prevalence of depression during the transitional period of early adulthood may contribute to improved outcomes throughout the lifespan. Those who experience depression during this transitional period are more likely to have recurrent depressive episodes during adulthood (5). Additionally, depressive symptoms during emerging adulthood may contribute to the erosion of personal and social resources (5) and affect socioeconomic outcomes (6,23). Depression during the transition to adulthood may interfere with establishing romantic relationships and identifying suitable career development and employment opportunities (24) as well as contribute to health risk behaviors (25). In light of the consequences related to experiencing depression and unemployment during this transitional period, it is important to develop interventions targeted to these young people. Early interventions within the population may not only ameliorate the negative effects of unemployment and depression among this group but also portend better mental health futures for them.

While this study has some strengths, including the use of the PHQ-8 as measure of depression (19) and the large and geographically diverse sample size, some limitations should be mentioned. The cross-sectional study design limits the ability to examine the causal relationship between unemployment and depression among emerging adults. Emerging adults who are depressed may have more difficulty finding and keeping a job. While most studies suggest that unemployment contributes to depression more than depression contributes to unemployment (8), we are unable to determine the direction of the relationship with these data. However, this study suggests that unemployed emerging adults are a population needing increased mental health care.

The generalizability of the study results is limited because of potential undercoverage of emerging adults in the BRFSS, because many of them use cellular telephones instead of landlines (26). In addition to undercoverage because of cellular telephone usage, the BRFSS sampling frame is household-based, which excludes those who live in institutions. The PHQ-8 questions were an optional module and only 12 states asked these questions in 2010. Therefore, the results are limited to respondents from those 12 states.

The main independent variable of interest, employment status, was based on the response to 1 question, where response options do not include answers that provide information about part-time employment or underemployment. Some individuals who were classified as employed may be working part time, which may also increase the risk of depression compared with full employment. With this categorization, the relationship between unemployment and depression may be underestimated (27). Additional information about the continuum of employment status would provide a more refined examination of the relationship between unemployment and depression. By refining the employment status along a continuum, analyses could compare unemployment and underemployment with full-time employment (27).

Including disability as a potentially confounding variable is another factor influencing the findings. The main disability question asks whether an individual is limited in any way because of physical, mental, or emotional problems. As mentioned, depression is a leading cause of disability (2), and individuals who are limited in their activities because of depression may give a positive response to this question. By controlling for disability in the second and third logistic regression models, the relationship between depression and unemployment was reduced. Therefore, the estimates presented probably understate the association between unemployment and depression.

While many emerging adults experience improved mental well-being during the transition to adulthood (5,11,28), numerous emerging adults experience depression during this time (23,29), especially those who are unemployed. We identify unemployment as a significant factor related to depression among unemployed emerging adults, but limited research is available that examines the effect of unemployment on depression among the emerging adult population. Therefore, research is needed to understand the experience of unemployment among emerging adults, especially research that examines differences from the experiences of unemployed adults who have been in the workforce for extended periods. Increasing understanding about the factors that contribute to the relationship between depression and unemployment among emerging adults could inform future preventive interventions and reduce the short-term and long-term consequences associated with unemployment among emerging adults. With the current high rates of unemployment for this age group, this population may benefit from interventions that specifically focus on their employment status and their mental health.
